# The Open Form Inducer Approach for Structure-Based Drug Design

**DOI:** 10.1371/journal.pone.0167078

**Published:** 2016-11-28

**Authors:** Daniel Ken Inaoka, Maiko Iida, Toshiyuki Tabuchi, Teruki Honma, Nayoung Lee, Satoshi Hashimoto, Shigeru Matsuoka, Takefumi Kuranaga, Kazuhito Sato, Tomoo Shiba, Kimitoshi Sakamoto, Emmanuel Oluwadare Balogun, Shigeo Suzuki, Takeshi Nara, Josmar Rodrigues da Rocha, Carlos Alberto Montanari, Akiko Tanaka, Masayuki Inoue, Kiyoshi Kita, Shigeharu Harada

**Affiliations:** 1 School of Tropical Medicine and Global Health, Nagasaki University, Nagasaki, Japan; 2 Department of Biomedical Chemistry, Graduate School of Medicine, The University of Tokyo, Tokyo, Japan; 3 Department of Integrated Analytical Chemistry, Graduate School of Pharmaceutical Sciences, The University of Tokyo, Tokyo, Japan; 4 Systems and Structural Biology Center, RIKEN, Tsurumi, Yokohama, Japan; 5 Department of Applied Biology, Graduate School of Science and Technology, Kyoto Institute of Technology, Kyoto, Japan; 6 Department of Biochemistry and Molecular Biology, Faculty of Agriculture and Life Science, Hirosaki University, Aomori, Japan; 7 Department of Molecular and Cellular Parasitology, Juntendo University School of Medicine, Tokyo, Japan; 8 Institute of Chemistry of São Carlos, University of São Paulo, São Paulo, Brazil; NCI at Frederick, UNITED STATES

## Abstract

Many open form (OF) structures of drug targets were obtained *a posteriori* by analysis of co-crystals with inhibitors. Therefore, obtaining the OF structure of a drug target *a priori* will accelerate development of potent inhibitors. In addition to its small active site, *Trypanosoma cruzi* dihydroorotate dehydrogenase (TcDHODH) is fully functional in its monomeric form, making drug design approaches targeting the active site and protein-protein interactions unrealistic. Therefore, a novel *a priori* approach was developed to determination the TcDHODH active site in OF. This approach consists of generating an "OF inducer" (predicted *in silico*) to bind the target and cause steric repulsion with flexible regions proximal to the active site that force it open. We provide the first *proof-of-concept* of this approach by predicting and crystallizing TcDHODH in complex with an OF inducer, thereby obtaining the OF *a priori* with its subsequent use in designing potent and selective inhibitors. Fourteen co-crystal structures of TcDHODH with the designed inhibitors are presented herein. This approach has potential to encourage drug design against diseases where the molecular targets are such difficult proteins possessing small AS volume. This approach can be extended to study open/close conformation of proteins in general, the identification of allosteric pockets and inhibitors for other drug targets where conventional drug design approaches are not applicable, as well as the effective exploitation of the increasing number of protein structures deposited in Protein Data Bank.

## Introduction

Pyrimidine nucleotides are DNA and RNA building blocks derived from *de novo* biosynthesis and/or salvage pathways. In humans, both pathways are functional and disruption of one can result in compensation by the other[1]. However, rapidly proliferating cells, including cancer cells or activated lymphocytes, rely on *de novo* biosynthesis for their growth[2]. Therefore, drugs targeting *de novo* biosynthesis, N-(phosphonacetyl)- L-aspartate (PALA) and leflunomide (Arava®, Sanofi-Aventis), have anti-cancer and immunosuppressive activities[3]^,^[2], respectively. PALA and A771726 (the active metabolite of leflunomide) inhibit the second (aspartate carbamoyltransferase) and fourth (dihydroorotate dehydrogenase, DHODH) steps in *de novo* biosynthesis, respectively.

In many pathogens, such as *Plasmodium falciparum* and *Helicobacter pylori*, the enzymes required for the salvage pathway are missing from their genomes[4]^,^[5], making them dependent on *de novo* biosynthesis. Among Trypanosomatid parasites, *Trypanosoma cruzi*, the pathogen of Chagas disease, is the only example which relies on pyrimidine *de novo* biosynthesis. Unlike *T*. *brucei* and *Leishmania* spp. (causative agents of human African trypanosomiasis and leishmaniasis, respectively), the intracellular stage of *T*. *cruzi* lacks uracil phosphoribosyltransferase enzymatic activity[6]. Additionally, another key enzyme in the pyrimidine salvage pathway (uridine kinase) is missing from the *T*. *cruzi* genome[7]. Consequently, pyrimidine *de novo* biosynthesis is not essential for *T*. *brucei* [8]^,^[9]^,^[10] and *L*. *donovani*[11] growth, while *T*. *cruzi* strictly depends on it[12]^,^[13]. Therefore, *de novo* pyrimidine biosynthesis is an attractive target for the development of new drugs to combat Chagas disease.

Among the six steps required for *de novo* biosynthesis, the fourth step is catalyzed by dihydroorotate dehydrogenase (DHODH) and is a promising drug target because of its diversity. DHODHs are classified into two families according to cellular localization and electron acceptor. Family 1 DHODHs are cytosolic enzymes found in gram-positive bacteria, archaea, and lower eukaryotes. They are further subdivided into family 1A, a homodimeric FMN-containing enzyme using fumarate as an electron acceptor, and family 1B, a heterotetrameric enzyme containing FMN, FAD, and [2Fe-2S] cluster and using NAD^+^ as an electron acceptor. Family 2 DHODHs are found in gram-negative bacteria and eukaryotes, and are localized in the plasma membrane (bacteria) or mitochondrial inner membrane (eukaryotes). They are monomeric/homodimeric enzymes containing FMN and utilize respiratory quinones as electron acceptors[14]. The fact that family 1A DHODH is not connected to the respiratory chain allows some organisms having this class of enzyme, such as Trypanosomatid parasites, being able to biosynthesize pyrimidine even in the hypoxic microenvironment[15]. *T*. *cruzi* DHODH (TcDHODH) belongs to family 1A and by using structures complexed with substrates and products, we previously showed that the enzyme utilizes the same site for the first-half (dihydroorotate oxidation) and second-half (fumarate reduction) reactions[16], while family 1B[17] and 2[18] utilize distinct sites for these reactions. However, there are no reports of a potent, specific inhibitors targeting family 1A DHODHs to date.

Respiratory chain (RC) enzymes are promising drug targets because of their wide diversity among species. For example, the RC of blood stream forms of *T*. *brucei* possesses trypanosome alternative oxidase (TAO), a membrane associated diiron protein that functions as the sole terminal oxidase, which is absent in human[19]. Potent inhibitor of TAO, ascofuranone (AF), has strong trypanocidal activity *in vitro* and *in vivo*[20]^,^[21]^,^[22]. Another example is an anaerobic RC of the helminthes. The RC of adult *Ascaris suum* and *Echinococcus multilocularis* protoscoleces, contain an anaerobic NADH-fumarate reductase (NADH-FRD) system composed of Complex I (NADH dehydrogenase)[23], low potential quinone species (rhodoquinone) and Complex II catalyzing the reverse reaction [quinol:fumarate reductase (QFR)[24]^,^[25]]. Inhibitors of Complex I have been shown to kill *E*. *multilocularis* protoscoleces *in vitro*, thus chemically validating the NADH-FRD system as a drug target[25]. We have also discovered atpenin A5 as the most potent inhibitor of respiratory Complex II which was later found to inhibit not only eukaryotic but bacterial enzyme as well [26]. Previously, we reported the crystal structures of TAO in complex with ascofuranone derivatives (3VVA and 3W54)[27] as well as *A*. *suum* QFR in complex with flutolanil (3VR9 and 3VRB)[28]^,^[29]^,^[30], its derivatives (4YSZ, 4YT0, 4YTM, 4YSX and 4YSY) and the substrate rhodoquinone (5C2T)[31], providing structural insights into their inhibition mechanisms. Both type of inhibitors bind to the quinol binding site of each target. Because family 2 DHODHs directly link pyrimidine *de novo* biosynthesis to the RC, structure-based drug-design (SBDD) targeting the ubiquinone binding sites of human[32]^,^[33]^,^[34] and *P*. *falciparum*[35]^,^[36]^,^[37]^,^[38] DHODHs have been extensively studied, and species-selective DHODH inhibitors were rationally designed. However, these inhibitors target the ubiquinone binding site of only family 2 enzymes and have no obvious effect on family 1A DHODHs since the later lacks the ubiquinone binding site. Because of differences in ubiquinone dependency between human and *T*. *cruzi* DHODHs, the main focus of this study was the design of potent and selective inhibitors targeting the family 1A enzyme TcDHODH.

The continuing discovery, in random screening studies, of potent inhibitors that bind and expand the volume (open form, OF) of the target molecule active site emphasizes the importance of the target protein OF structure in drug discovery[39]. However, all targetable OF structures reported to date were obtained *a posteriori* to the discovered inhibitors. Specifically, they were unexpectedly found binding to the OF after determination of target/inhibitor co-crystal structures. Thus, we hypothesized that obtaining the OF structure of a target protein *a priori* will enable the design of potent inhibitors using conventional approaches. However, the lack of tools and reports where the OF structure of a target protein was rationally obtained *a priori* have substantially delayed structure based drug design (SBDD) targeting TcDHODH.

In this study, the “OF inducer” approach was developed in order to obtain the OF structure of TcDHODH *a priori*. Here, the OF inducer was designed and co-crystallized with TcDHODH. As expected, the structure of TcDHODH with an OF active site was successfully determined. The OF structure was subsequently used to design and synthesize several potent and selective inhibitors. From the 13 orotate derivatives designed in this study, the co-crystal structures of TcDHODH in complex with all derivatives were obtained. This allowed us to validate the OF inducer approach as a novel and powerful tool to understand the OF structures and consequently enabling the design of potent and specific inhibitors.

## Material and Methods

### Enzyme assays

The enzyme activity of TcDHODH was assayed spectrophotometrically by monitoring the direct production of orotate at 290 nm. The TcDHODH standard assay (TSA) solution contained 100 mM sodium phosphate buffer (pH 8.0), 2 mM fumarate, and 1 μg/ml of TcDHODH. Equal volumes (2 μl) of an inhibitor dilutions series (11 wells for inhibitor plus 1 for DMSO control) were transferred to a 96 well plate with UV transparent flat bottom (Corning Inc.) followed by 188 μl of TSA solution. To assess background activity, the plate was placed in a SpectraMax M2e-TUY plate reader (Molecular Devices), mixed for 5 seconds before the first read (kinetic mode) and 3 seconds between reads during the 5 minute assay. The reaction was started by quickly adding 10 μl of 10 mM L-dihydroorotate stock solution (final concentration of 500 μM) and TcDHODH activity was assayed with the same parameters used in the background assay. The rate (Abs/min) during the first 2 minutes was calculated using SoftMax Pro 5.3 software (Molecular Devices). The rate relative to DMSO control (100%) versus inhibitor concentration was plotted and IC_50_ values calculated as the concentration of inhibitor that produces a 50% reduction in relative enzymatic activity. For HsDHODH, the standard assay (HSA) mixture contained 100 mM HEPES (pH 8.0), 150 mM NaCl, 5% (v/v) glycerol, 0.05% (w/v) Triton X-100, 200 μM L-dihydroorotate, 120 μM 2,6-dichloroindophenol (DCIP), and 14 μM decylubiquinone (dUQ). A reaction start (RS) solution was prepared, containing 40 μg/ml HsDHODH (specific activity of 50 μmol/min/mg) in 100 mM HEPES (pH 8.0), 250 mM NaCl, 5% (v/v) glycerol, and 0.05% (w/v) Triton X-100. Ten microliter of inhibitor dilution series were transferred to a 96 well plate (Nunc) and mixed with 185 μl of HSA. The plate was vortexed for 20 seconds and the background recorded for 5 min at 600 nm. The reaction was started by adding 5 μl of RS solution, vortexing for 20 seconds, and HsDHODH activity assayed using the end point method after 20 minutes with a SpectraMax M2e-TUY plate reader (Molecular Devices) and analyzed using SoftMax Pro 5.3 software (Molecular Devices). The amount of DCIP reduced in the presence of DMSO was set to 100%, the relative activity versus inhibitor concentration was plotted and the IC_50_ values calculated. Each data point was assayed in triplicate on the same day. *K*_i_^app^ values were calculated using the Cheng-Prusoff equation with a *K*_m_ for dUQ of 14.0 μM and for dihydroorotate of 25.9 μM as previous described, for HsDHODH[33] and TcDHODH[15], respectively.

### Size exclusion chromatography

The size of TcDHODH was estimated by HPLC using a TSK gel G3000SW (Tosoh) column (7.5 × 600 mm), as previously described[15]^,^[16]. Purified TcDHODH (0.5 mg) was mixed with 50 μl of Gel Filtration Standard (Bio-Rad) and injected to the column. The analysis was performed at room temperature with a flow rate of 1 ml/min in 100 mM sodium phosphate buffer (pH 7.5) containing 500 mM NaCl and 0.25 mM sodium orotate. The molecular weight of TcDHODH was calculated as 37 kDa based on the retention times of proteins in the Gel Filtration Standard (Bio-Rad).

### DHODH activity staining

Blue native PAGE (BN-PAGE) of TcDHODH was performed according to the manufacturer’s instructions. 1250 to 20 ng of pure TcDHODH in 5% (v/v) glycerol and 0.01% (w/v) Ponceau S was loaded onto a 4–16% (w/v) Bis-Tris gel (Invitrogen) and run at 150 V using the light blue cathode buffer method [0.002% (w/v) of G-250 blue dye] at 4°C. Electrophoresis was terminated when the Ponceau S band reached approximately 1 cm from the bottom of the gel. The gel was transferred to a container, washed three times with 5 mM Tris-HCl pH 7.4 and incubated with 5 mM Tris-HCl pH 7.4 containing 2.5 mg/ml nitro blue tetrazolium chloride. The TcDHODH band was stained with L-dihydroorotate (0.25 mM) without phenazine methosulfate (PMS) at room temperature. A dark purple band corresponding to TcDHODH appeared within 5 seconds in the lane containing 1250 ng and within 2 minutes in the lane containing 20 ng. Since HsDHODH is a membrane protein and requires Triton X-100, high-resolution clear native electrophoresis was chosen for DHODH activity staining. Purified HsDHODH (1250 to 50 ng) was loaded onto 4–16% (w/v) Bis-Tris gel (Invitrogen) and run at 150 V at 4°C, according to the manufacturer’s instructions. HsDHODH activity was stained as described for TcDHODH, however in the presence of 0.06 mg/ml PMS. After DHODH activity staining, the gels were extensively washed with water and the molecular weight marker (NativeMark^TM^ Unstained Protein Standard, BioRad) was stained with GelCode® Blue Stain Reagent (Pierce). The molecular weight of TcDHODH and HsDHODH was calculated by constructing a Ferguson plot[40] using the NativeMark^TM^ standards.

### Assessment of steric repulsion between 5-substituted orotate and the active site loop moiety

**Fig 1:**
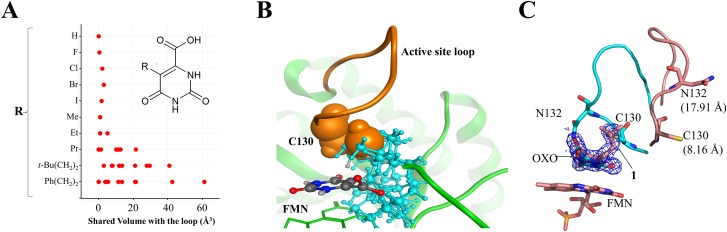
Generation of OF inducer. (A) Shared volume between various conformers of the 5-substituent group (inset) and C130 from the active site loop. The graph was generated using coordinates from the TcDHODH-orotate complex structure (2E6A)[16] and calculated using MOE[41]. (B) Superposition of various conformations of **1** generated by MOE[41] to the active site of the TcDHODH-orotate complex structure. FMN and TcDHODH are shown in green as stick and ribbon, respectively. The active site loop and C130 are shown in orange. Different conformations of **1** are shown as ball-and-stick with carbon, nitrogen and oxygen colored in gray, blue, and red respectively. For better visualization, all atoms from the *t*-butyl-ethyl moiety are colored in cyan. (C) Superposition of the B chain from TcDHODH-1 (3W1Q, salmon) and -oxonate (2E6F, cyan)[16] complex structures. The electron density map, contoured at 1 σ, from **1** is shown. C130 and N132 residues are represented as stick. The distance moved relative to the respective positions from the TcDHODH-oxonate complex structure (2E6F)[16] for Sγ group (C130) and Nδ^2^ (N132) are shown in parenthesis.

To obtain a crystal structure with open loop conformation, steric repulsion between substituents (R-groups) at the 5-position of orotate and the active site loop (L128-Q138) of the closed form of TcDHODH were assessed. Firstly, nine orotate derivatives with different sized 5-substituents were prepared *in silico*. The conformational searches of the nine derivatives were performed using the modeling software MOE 2007.09[41], with MMFF94x force field, and stable conformers (ΔE ≤ 5 kcal/mol) for each compound were selected. The generated conformers were superposed with orotate on the closed form of TcDHODH. Using the superposed models, van der Waals volumes shared between a substituent at the 5-position of orotate and the active site loop was calculated for each conformer. Fig 1A shows the distribution of the calculated shared volumes of the orotate derivatives. To stably change the conformation of the loop, propyl, 3,3-dimethylbuthyl, and ethylbenzene groups were predicted to have sufficient sizes compared with those of halogen, methyl, and ethyl groups. Orotate derivative containing 3,3-dimethylbuthyl was selected as the first OF inducer and its binding mode confirmed by co-crystallographic study.

### Design of the 5-substituted orotate library based on the open form of TcDHODH

From the analysis of ligand contact preference around the orotate binding site according to the modeling software MOE[41], the binding region around the 5-position of orotate was predicted to preferably interact with hydrophobic ligand atoms. In fact, the region is surrounded by the hydrophobic amino acid residues L71, L101, and P131. Across the hydrophobic region, there are H-bonding sites, such as N53 and R50, which are accessible with substituents at the 5-position of orotate. To improve potency of orotate derivatives by interacting with the predicted pharmacophore features, we designed a 5-substituted orotate library. 5-Substituted orotate derivatives can be synthesized using a coupling reaction between 5-bromoorotate and tributylstannyl olefins. Tributylstannyl olefins can be prepared from terminal alkynes. The 2170 commercially available terminal alkynes listed in the ACD database (Molecular Design Limited, San Leandro, CA, USA) were used to construct a virtual 5-substituted orotate library. The terminal alkynes were transformed into their 5-substituted orotate forms using a customized protocol on Pipeline Pilot[42]. The 2170 virtual compounds were docked with the open form of TcDHODH using the docking program GLIDE[43]. The crystal structure of the TcDHODH-**1** complex has two chains with different degrees of openness. Between the two chains, the more open form of TcDHODH was used for docking. From the docking results of the 2170 compounds, 225 compounds with new interaction(s) and good docking scores (GLIDE score ≤ -10.0 kcal/mol) were selected; the GLIDE score criteria (-10.0 kcal/mol) was determined according to the score distribution of known TcDHODH inhibitors such as orotate, oxonate, and succinate. The 225 compounds were clustered into three groups using interaction pattern analysis. Higher scoring compounds from each cluster (9 compounds in total) were identified and selected for synthesis.

### Synthesis of 5-substituted orotate derivatives

Alkyne (1.5 mmol), PdCl_2_(PPh_3_)_2_ (0.1 mmol), CuI (0.2 mmol), and Et_3_N (2 mmol) were added to an iodide [CAS: 116393-71-6] (1 mmol) solution in THF (5 mL) at room temperature. After stirring overnight at room temperature, the mixture was diluted with hexane and filtered. The residue was washed with CH_2_Cl_2_ and dried *in vacuo* to afford crude alkyne, which was used in the next reaction without further purification.

Pd/C [(5% (w/w)] or Pd(OH)_2_/C [20% (w/w)] was added to a solution of the crude alkyne in MeOH/AcOH (9:1, 10 mL). After stirring under H_2_ atmosphere at room temperature for 12 h, the mixture was filtrated through a celite pad. The residue was washed with MeOH and dried *in vacuo* to afford crude methyl ester, which was used in the next reaction without further purification.

1 M NaOH aq (0.1 mL) was added to a suspension of the crude methyl ester in EtOH (1 mL) at room temperature. After stirring at 50°C for 6 h, the solvent was evaporated. The resulting solid was dissolved in monoethanolamine-AcOH (pH 9) buffer and purified with HPLC (monoethanolamine-AcOH (pH 9) buffer/CH_3_CN) to afford the orotic acid derivatives as monoethanolamine salts.

### Crystallization

TcDHODH was expressed, purified, and crystallized essentially according to the methods described previously by Inaoka[44]^,^ [16]. Co-crystal structures of TcDHODH with 5-substituted orotate derivatives were prepared by initial soaking of TcDHODH-oxonate crystals in a fresh reservoir solution containing 0.05 mM 5-substituted orotate derivatives (soaking solution) and then gradually increasing its concentration every 12 hours, up to 0.2 ~ 5 mM.

### Data collection

The TcDHODH crystal was briefly soaked in the reservoir solution with highest concentration of 5-substituted orotate derivatives containing 20% (v/v) glycerol and flash-frozen in a nitrogen stream at the beam line [Spring-8 (SP8) beam lines: BL44XU and BL41XU; Photon Factory (PF) beam lines: BL5A, BL17A, NE3A, or NW12A; see S1 Table]. All the data sets were processed and scaled with HKL2000[45].

### Refinement

The crystal structure of TcDHODH in complex with compound **1** was solved by molecular replacement using the coordinates from TcDHODH-oxonate (2E6F)[16] as the search model. For the remaining co-crystal structures of TcDHODH in open form, the coordinates from TcDHODH-compound **1** (3W1Q) was used. Manual building of the remaining model from TcDHODH and further crystallographic refinement were performed with the COOT[46] and REFMAC5[47] softwares. Data collection and structural refinement statistics are summarized in S1 Table. Figures showing protein structures were prepared with the graphics programs PyMol (http://www.pymol.org/) and MOE[41].

## Results and discussion

### Open form inducer design

**Fig 2:**
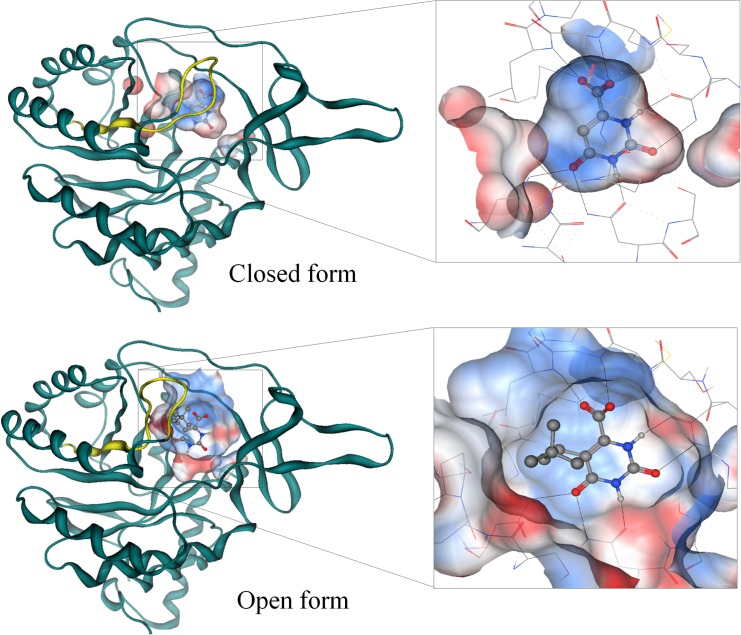
The active site structure upon binding of OF inducer. (Top) Ribbon representation of the TcDHODH structure in complex with orotate (ORO, PDB: 2E6A)[16]. (Bottom) Structure of the TcDHODH in complex with **1** (3W1Q). Yellow loop represents the active site loop (S128—V139). A “zoom in” pose of the active site pocket is shown (inset) with hydrogen bonds represented as dotted lines. Ball-and-stick represents ORO and atoms of carbon, nitrogen, oxygen, and hydrogen colored in gray, blue, red, and white, respectively.

The small volume of the closed form of TcDHODH impedes the design of nanomolar inhibitors. Therefore, a new approach was developed to obtain the OF *a priori* for the purpose of designing potent inhibitors. In the ligand-free structure of TcDHODH, the electron density map of the active site loop (L128-D142) is poorly defined, indicating a high degree of flexibility[16], which is stabilized only by binding of ligands in the active site. We have previously reported that the Sγ atom of C130 in the active site loop, the key residue during catalysis, is located just 3.52 Å away from C5 of the dihydroorotate pyrimidine ring[16]. Because of the close contact with the active site loop, several orotate derivatives containing small substituents at C5 of the pyrimidine ring were analyzed *in silico*. The degree of steric repulsion with the flexible loop was calculated as the shared volume between C130 and various conformers of 5-substituents (Fig 1A). Of the 5-substituted derivatives analyzed, the *t*-butyl-ethyl and phenyl-ethyl groups were predicted to cause higher degrees of steric repulsion than halogen and short alkyl groups (Fig 1A). Fig 1B shows the calculated alternative conformers of **1**, docked into the orotate binding site, causing steric repulsion with C130. To validate the OF inducer approach, **1** was synthesized and evaluated. Despite the low inhibitory activity of **1** (IC_50_ ≥ 400 μM, Table 1), the crystal structure of TcDHODH in complex with **1** was successfully prepared by soaking method. After refinement, a clear electron density map corresponding to **1** was readily distinguished from oxonate (Fig 1C). As expected, **1** was found binding with its *t*-butyl-ethyl group replacing the position occupied by Sγ of C130 in the closed form, forcing active site opening (Fig 1C). Previously, three regions around the active site loop were identified as potential drug-binding sites: the active site (S1), extension of S1 site (S2), and the site behind the active site loop (S3)[48]. The binding of **1** pushed the active site loop to the S2 site; in addition, the S1 and S3 sites become connected by the region previously occupied by the active site loop (S1 Fig). Binding also increased to more open in the active site of chain B than A due to crystallization packing. In chain B, Sγ of C130 moved 8.16 Å while Nδ of N132 moved 17.91 Å (Fig 1C). The drastic movement of the active site loop in chain B expanded the active site volume from 178 Å^3^ (closed form, Fig 2 top) to 694 Å^3^ (OF, Fig 2 bottom). The OF active site completely revealed the tunnel connecting the solvent to the active site. Binding also exposed the hydrophobic region and hydrogen bonding sites located across the binding site which are predicted to be accessible with 5-substitutions of the orotate pyrimidine ring (S2 Fig).

**Table 1:** 5-substituted orotate derivatives designed in this study.

Inhibitor	5-substituent	TcDHODH (μM)		HsDHODH (μM)		SI	Co-crystal^b^	
		IC_50_	*K*_i_^app^	IC_50_	*K*_i_^app^	(Fold)^a^	Resolution (Å)	PDB^c^
ORO^d^	H	112 ± 21	5.51	n.d.^e^	n.d.	n.d.	1.64	2E6A
**1**	2,2‐dimethylpentane	> 400	> 19.7	n.d.	n.d.	n.d.	1.85	3W1Q
**2**	propylbenzene	62.4 ± 16.9	3.07	> 5,000	> 2,500	> 814	1.58	3W1R
**3**	1-propyl-3-(trifluoromethyl)benzene	12.5 ± 0.8	0.62	284 ± 50	142	231	1.68	3W1T
**4**	2-propylnaphthalene	4.51 ± 0.09	0.22	46.1 ± 17.2	23.1	104	1.67	3W7H
**5**	1-propylnaphthalene	15.5 ± 0.8	0.76	434 ± 41	217	284	1.55	3W72
**6**	1-methoxy-6-propylnaphthalene	2.24 ± 0.42	0.110	20.6 ± 3.1	10.3	93.6	1.75	3W7C
**7**	2-methoxy-6-propylnaphthalene	2.89 ± 0.07	0.14	46.2 ± 3.2	23.1	163	1.97	3W3O
**8**	2-methoxy-7-propylnaphthalene	5.90 ± 0.19	0.29	13.3 ± 0.5	6.65	22.8	1.82	4JDB
**9**	6-propylnaphthalene-1-carboxylic acid	0.67 ± 0.06	0.03	> 5,000	>2,500	> 75,760	2.63	3W6Y
**10**	6-propylnaphthalene-2-carboxylic acid	0.50 ± 0.03	0.02	1,785 ± 1,773	893	37,210	1.58	3W7J
**11**	1-methyl-4-propylbenzene	8.68 ± 0.68	0.427	5.77 ± 0.66	2.88	6.75	1.85	3W1U
**12**	1-tert-butyl-4-propylbenzene	22.4 ± 2.1	1.10	16.8 ± 1.4	8.40	7.64	1.98	3W22
**13**	1-phenoxy-4-propylbenzene	22.6 ± 1.5	1.11	16.6 ± 1.8	8.30	7.48	2.60	3W70
**14**	4-propyl-1,1'-biphenyl	16.0 ± 1.9	0.79	0.97 ± 0.18	0.48	0.61	1.68	3W71

^a^, selectivity index calculated from the ratio of HsDHODH *K*_i_^app^ / TcDHODH *K*_i_^app^ values.

^b^, information from co-crystal structures of 5-substituents with TcDHODH.

^c^, Protein Data Bank identification code

^d^, orotate

^e^, not determined. *K*_i_^app^ was calculated from IC_50_ values according to Material and Methods. For detailed crystallization statistics see S1 and S2 Tables.

### Design of potent and selective 5-substituted orotate derivatives

**Fig 3:**
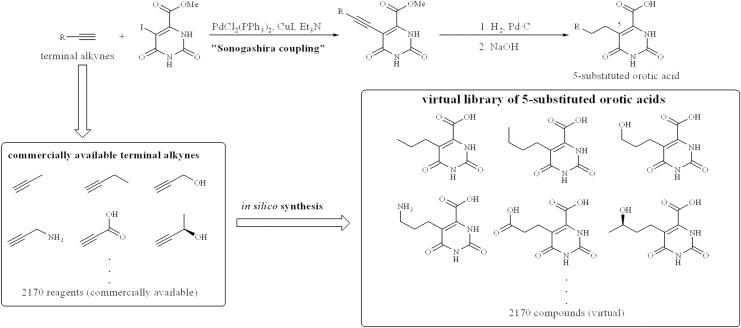
Virtual library of 5-substituted orotate derivatives. 5-Substituted orotate derivatives can be synthesized by Sonogashira coupling using terminal alkynes. A virtual library was generated *in silico* using all 2170 commercially available terminal alkynes, screened against the more OF TcDHODH-**1** crystal structure and clustered into groups according to their docking scores. 20 compounds with high docking scores were selected and synthesized.

5-Substituted orotate derivatives can be easily synthesized from terminal alkynes (Fig 3). Using commercially available terminal alkynes, a virtual library containing 2170 orotate derivatives was constructed and docked into the OF TcDHODH (Fig 3). From the resulting 225 compounds with high docking scores, 4 compounds (**2**–**5**) with higher docking scores, predicted to interact with the hydrophobic region, were selected and synthesized (Table 1). To validate our predictions, the IC_50_ and *K*_i_^app^ values of **2**–**5** were analyzed. All 4 compounds were more potent than orotate (Table 1). In order to determine the selectivity index (SI) of the orotate derivatives, recombinant human DHODH (HsDHODH) was purified and the IC_50_ and *K*_i_^app^ values determined. **2** did not inhibit HsDHODH at high concentrations (5 mM) and exhibited higher SI (> 814-fold) than **3**, **4**, and **5** (231, 104, and 284, respectively; Table 1). To confirm the structural basis of predicted *versus* crystallographic inhibitor binding modes and selectivity, the structures of TcDHODH complexed with **2**–**5** were determined. The binding mode of each derivative was found to be consistent with our docking model (S3 Fig). In the crystal structures, the 5-substituent group of **2**–**5** (Table 1) interacts through CH-π interactions with P131 at the hydrophobic region, which explains their increased inhibitory potency compared to orotate (S3 Fig). The low *K*_i_^app^ (0.22 μM) of **4** is the result of a unique interaction, in which the naphthyl group is sandwiched by CH-π interactions between G70 and P131, which are not observed with compounds **2**, **3**, and **5** (S3 Fig). The high selectivity of those compounds against TcDHODH is attributed to the L71 to F149 substitutions in HsDHODH. The bulkier side chain of F149, compared to L71, causes steric repulsion with the 5-substituted group, making binding to HsDHODH unfavorable. From these results we can conclude that the hydrophobic region can be targeted to increase the inhibitory potency as well as to increase the selectivity of these compounds toward TcDHODH.

The effect of an additional hydrogen bond in the 5-substituted orotate derivatives was also investigated by introducing methoxy groups at positions 5, 6, and 7 of the naphthyl ring (**6**, **7**, and **8**, respectively, Table 1). **6** and **7** decreased the by 2-fold (0.11 μM and 0.14 μM, respectively, Table 1), and *K*_i_^app^ is slightly increased in **8** (0.29 μM), in comparison to **4** (0.22 μM). These changes in *K*_i_^app^ values can be clearly explained by the co-crystal structures. According to our predictions, the methoxy groups on **6**, **7**, and **8** should form hydrogen bonds with the NH_2_ side chain of N53. In fact, a hydrogen bond between the methoxy group and N53 was observed in **7** bound to the A and B chains and in **8** bound to the A chain. However, an unexpected hydrophobic interaction between the methoxy group and the alkyl side chain of K214 was found in **6** (S4 Fig). This hydrophobic interaction was also seen in the A chain of the TcDHODH-**7** co-crystal structure. The comparison of binding modes and *K*_i_^app^ (Table 1) between **6** and **7** clearly indicates that the loss of the hydrogen bond with N53 can be compensated with an unusual hydrophobic interaction with the alkyl side chain of nearby K214 without affecting the *K*_i_^app^ values. Since the CH-π interaction between P131 and the naphthyl group of **8** was lost (S4 Fig), this may contribute to the slightly higher *K*_i_^app^ value of **8** compared to **6** and **7**. These results indicate that optimum interaction with K214 and N53 can be achieved by introducing a hydrogen bond acceptor at positions 5 and 6 of the naphthyl group, respectively.

In order to strengthen the interaction with K214 and N53, carboxylate groups at positions 5 and 6 (**9** and **10**, respectively, Table 1) of the naphthyl group were introduced. Considering the p*K*_a_ of 3.69 and 4.17 of 1- and 2-naphthoic acid, respectively[49], it is expected that the carboxylate groups in **9** and **10** should be present in anionic form during crystallographic studies (pH 5.3). Carboxylate group substitution remarkably decreased the *K*_i_^app^ of **9** (0.03 μM) and **10** (0.02 μM). It also increased the *K*_i_^app^ for HsDHODH to >2500 and 893 μM for **9** and **10**, respectively (Table 1). Consequently, this dramatically increased the selectivity index against TcDHODH to >75800 and 37200 for **9** and **10**, respectively (Table 1).

**Fig 4:**
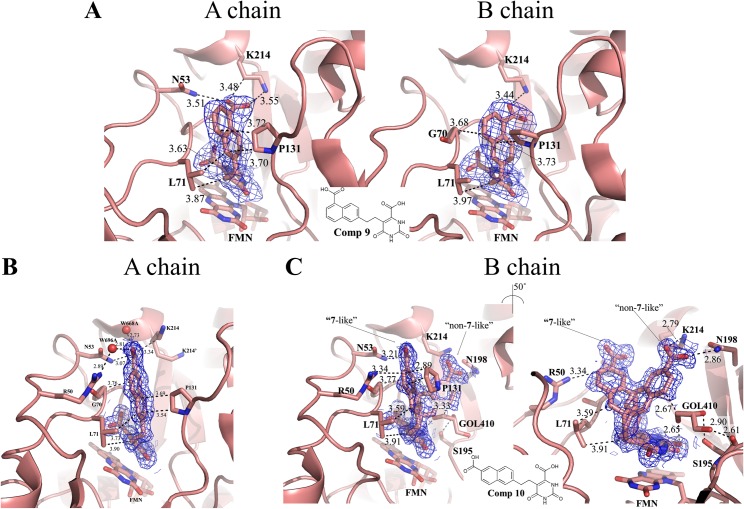
Binding modes of the top 2 TcDHODH inhibitors designed in this study. (A) Binding mode of **9** to chains A (left) and B (right); (B) Binding mode of **10** to the A chain; (C) Dual binding mode of **10** into the B chain. Left and right panels emphasize the interactions of 5-substituent groups from **7**-like and non-**7**-like binding modes, respectively. Key residues within a 4.0 Å distance to 5-subtituents **9**, **10**, and FMN are shown as stick. Dashed line represents hydrophobic or hydrogen bond interaction. Atoms of carbon, nitrogen, and oxygen are colored in salmon, blue, and red, respectively. Electron density maps are shown as blue mesh and contoured at a 1 σ level.

Since **9** and **10** are the most potent inhibitors of family 1A DHODHs reported, their co-crystal structures in complexes with TcDHODH were obtained and the binding mode was analyzed in detail. Fig 4A shows the binding mode of **9** in the A (left) and B (right) chains of TcDHODH. As expected, the carboxylate group of **9** formed a salt bridge with K214. With **10**, the predicted hydrogen bond with N53 in chain A was confirmed by the co-crystal structure (Fig 4B). Interestingly, **10** was found binding in a dual mode in the B chain (Fig 4C); “**7**-like” and “non-**7**-like” binding modes perfectly fit into the density map (Fig 4C). In **7**-like mode, the carboxylate group forms a hydrogen bond with N53 (Fig 4C, left) while in non-**7**-like mode it forms a salt bridge with K214 and a hydrogen bond with N198 (Fig 4C, right). The similarity of the *B*-factors of the non-**7**-like (9.2) and **7**-like (10.2) binding modes of **10** and the protein [*B*-factors are 9.8 and 11.8 for the A and B chains, respectively (S1 Table)] indicates that both conformations of **10** are stably bound to TcDHODH. Moreover, a cryoprotectant glycerol molecule (GOL410) was found interacting with the naphthyl ring of the non-**7**-like mode and the O2 orotate pyrimidine ring of **10** (Fig 4C, right) in the B chain. In addition, GOL410 forms hydrogen bonds with the side chain hydroxyl group and main chain oxygen of S195 (Fig 4C, right). This finding indicates the possibility of developing more potent orotate derivatives using a fragment-based approach, which is now in progress.

### Para-alkyl-phenylethyl orotate derivatives as novel Q-site inhibitors of HsDHODH

**Fig 5:**
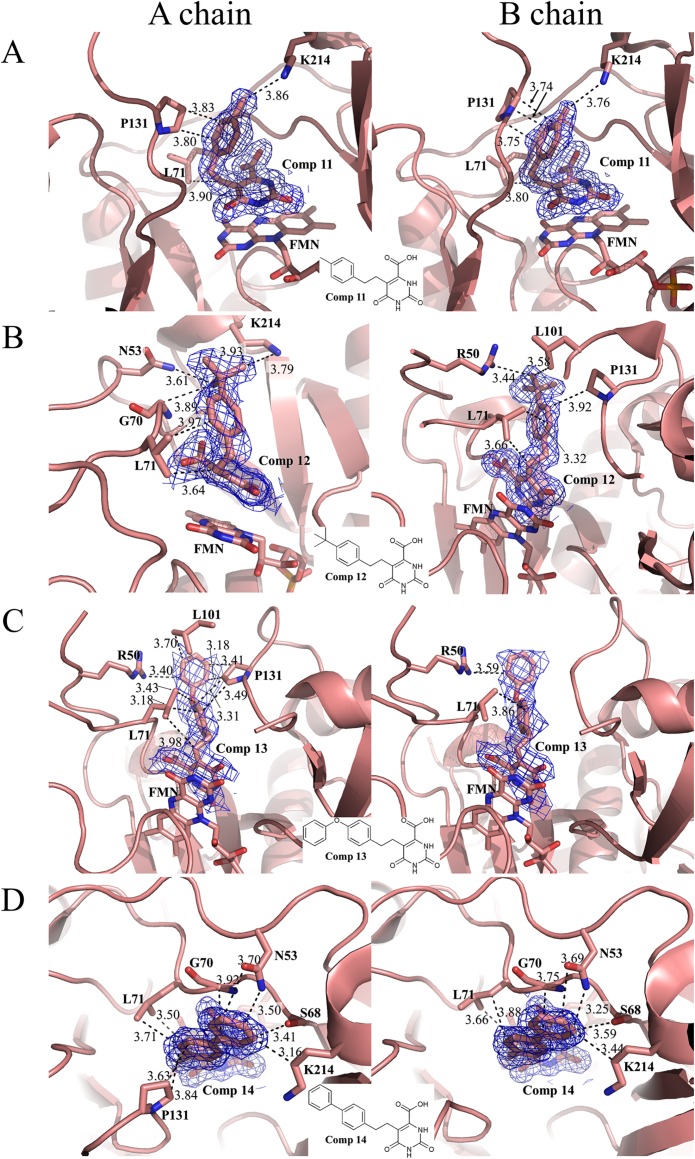
**Binding mode of Comp 11 (A), 12 (B), 13 (C) and 14 (D) into chains A (left) and B (right) of TcDHODH.** Key residues within 4.0 Å distance to 5-subtituent groups, inhibitors and FMN are shown as stick. Dashed lines represent hydrophobic or hydrogen bond interactions between 5-substitutent and protein residues within 4.0 Å distance, which are shown as the number next to the respective interactions. Color codes are same as Fig 4. Electron density map of Comp **11**–**14** are shown as blue mesh and contoured at 1 σ level.

**Fig 6:**
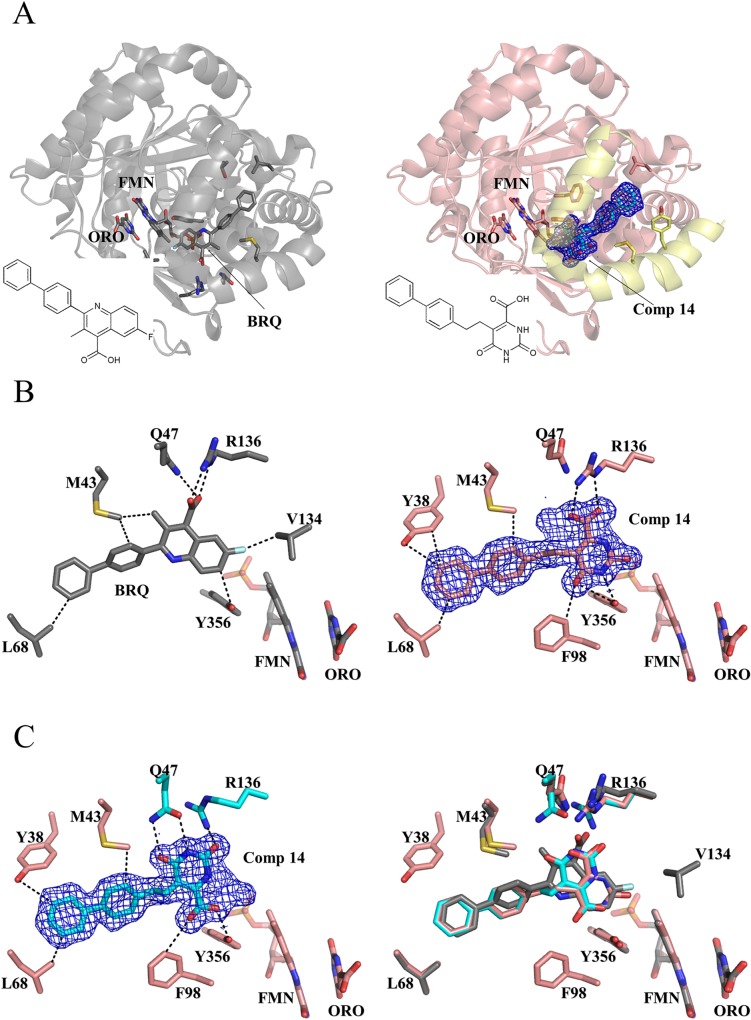
Overlapping binding site between brequinar analog and 14. (A) Overall structure of HsDHODH complexed with brequinar analog (BRQ, left, gray, 1D3G) [18] and **14** (right, pink, 3W7R). The N-terminal region, which attaches the protein to the mitochondrial inner membrane and proposed to be the ubiquinone reduction site, is highlighted in yellow. (B) Binding modes of the brequinar analog (left, 1D3G)[18] and **14** (right). BRQ, **14**, FMN, ORO, and key residues are shown in stick. Interactions within 3.5 Å are shown as dashed lines. Note the interaction of the carboxylate group from **14** with R136 (right), as in the BRQ analog (left), resulting in a “brequinar-like” binding mode. (C) The carboxylate group of **14** interacts with Y356 in a “non-brequinar-like” binding mode (left). BRQ and the dual binding modes of **14** were superposed in the right figure. Color codes for nitrogen, oxygen, sulfur, and fluorine are blue, red, dark yellow, and white, respectively. Carbon atoms from BRQ analog and **14** bound in a brequinar-like mode are gray and salmon, respectively. Carbon atoms from **14** and the two residues (Q47 and R136) that change their conformation in a non-brequinar-like binding mode are colored in cyan. The electron density map of **14** is shown as blue mesh and contoured at a 1 σ level.

During the drug-design process, we found that four 5-phenylethyl orotate derivatives containing an alkylated substitution at the *para* position of the phenyl ring (**11**–**14**, Table 1) exhibited increased inhibition of HsDHODH (Table 1). Co-crystal structures show that **11**–**14** bind to the TcDHODH OF and interact with the hydrophobic region, as expected (Fig 5A to 5D). Because of the L71 to F149 substitution in HsDHODH, **11**–**14** were not predicted to inhibit HsDHODH. However, all four compounds showed remarkably decreased *K*_i_^app^ against HsDHODH. In particular, **14** shows a *K*_i_^app^ of 0.485 μM for HsDHODH and a very poor selectivity index (0.61). As a positive control for HsDHODH assays, the active metabolite of leflunomide, A771726, exhibited an IC_50_ of 0.38 μM (*K*_i_^app^ = 0.19 μM) in our assay conditions, consistent with the reported *K*_i_ value of 0.18 μM[50]. To understand the structural basis of HsDHODH inhibition by **11**–**14**, we obtained the co-crystal structure of **14** with HsDHODH and compared it to that of TcDHODH (S2 Table). Surprisingly, **14** did not bind to the orotate binding site. Instead, it was bound to the hydrophobic tunnel involved in the binding of ubiquinone (Q-site) at the N-terminal region (Fig 6A), which is absent in TcDHODH.

Brequinar is a competitive inhibitor versus ubiquinone[51] for rat DHODH and the crystal structure of a brequinar analog-HsDHODH complex (Fig 6A) is available (1D3G)[18]. Comparison of crystal structures of HsDHODH in complex with **14** (Fig 6A, right) and a brequinar analog (Fig 6A, left) clearly shows that the binding sites of these inhibitors completely overlap (Fig 6B, left and Fig 6C, right). Interestingly, **14** bound the Q-site in dual mode, which are brequinar-like (Fig 6B, right) and non-brequinar-like (Fig 6C, left). In brequinar-like mode, the carboxylate group of **14** interacts with R136 (Fig 6A, right), while interacting with Y356 in non-brequinar-like mode (Fig 6B, left). This versatile property of the Q-site of HsDHODH, allowing binding of different conformations of carboxylated inhibitors, is consistent with the observations of Baungartner and colleagues[34]. Because of the structural similarity of **11**–**14**, we can conclude that the decreased specificity is the result of binding at the Q-site and not to the orotate binding site of HsDHODH.

### Correlation between docking scores and IC_50_

Finally, correlation analysis between *in silico* and inhibition studies of all 5-substituted orotate derivatives was performed (S5 Fig). The result clearly show that a good correlation between calculated docking scores and experimentally determined IC_50_s for all compounds designed in this study, thus validating our drug design methodology.

## Conclusion

This study provides the first proof-of-concept for a rational determination of protein OF conformations *a priori* using the OF inducer approach, specifically for proteins containing flexible regions around their active site. We also show that determination of the OF structure at the early stage of SBDD constitutes a shortcut for obtaining nanomolar inhibitors.

**Fig 7:**
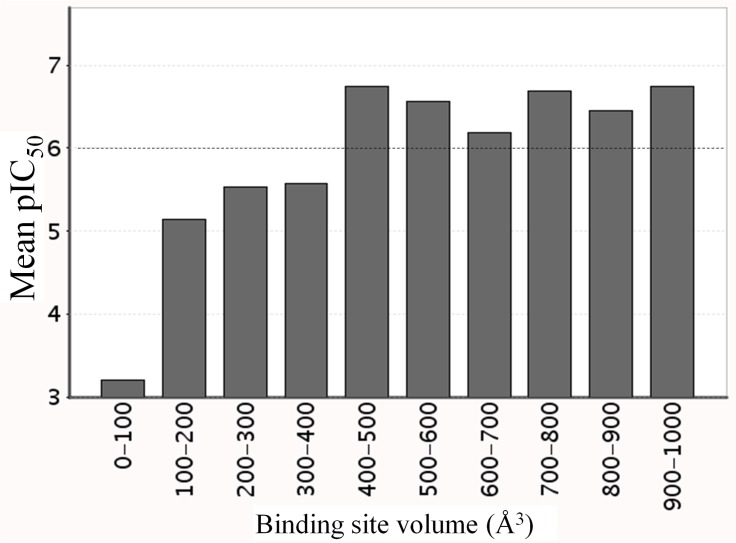
Histogram correlating the volume of inhibitor’s binding site versus pIC_50_, generated using ca. 3000 inhibitor/target complex structures with known IC_50_s from PDBbind data base. Dashed line represents the threshold between micromolar and nanomolar order inhibitor.

The X-ray analyses of ligand-free TcDHODH structures, as well as in complex with ligands, show that the L128-D142 loop functions as a lid, completely covering the active site (closed form). *In silico* screening based on the active site structure in the closed form failed to identify lead compounds that inhibit TcDHODH more potently than orotate (unpublished results). To explain this, a histogram correlating the inhibitor binding site volume versus IC_50_ was generated using over 3125 inhibitor-target complexes with known IC_50_s (from PDBbind). Binding site volumes > 400 Å^3^ are necessary to design inhibitors with nanomolar IC_50_ values (Fig 7). Because of the small volume (178 Å^3^) of the TcDHODH closed form active site, the design of inhibitors targeting the active site was unrealistic.

Successful examples of SBDD targeting protein-protein interaction (PPI) sites (ex. Bcl-2 family proteins)[52]^,^[53]^,^[23], allosteric sites (ex. G-protein-coupled receptor)[54], or active sites (ex. human and *P*. *falciparum* DHODHs)[33]^,^[55] have been reported. The crucial prerequisite for targeting PPI sites is the necessity of protein subunit association for biological function in the target protein. Enzymes belonging to family 1A are homodimeric and inhibitors targeting the interface between monomers may be attractive sites for drug design. However, evidence from size-exclusion chromatography, activity staining and dynamic light scattering indicate that the PPI approach was not applicable to TcDHODH, which functions in monomeric form and becomes homodimeric at high concentrations only (S6 and S7 Figs and S3 Table).

Difficulties in obtaining the OF structure of a target protein with the small volume of a drug binding pocket, are common problems encountered during drug-design. In this study we show that TcDHODH has a small active site and is the first monomeric enzyme among reported family 1A DHODHs. From a practical standpoint, conventional approaches for drug design were not applicable for TcDHODH. We have previously reported that TcDHODH contains a highly flexible active site loop composed of residues from L128 to D142, as demonstrated by the ligand-free structure[16]. Additionally, the co-crystal structures with different ligands could be easily obtained by soaking method. This has made TcDHODH an ideal model to design potent inhibitors using the OF inducer approach.

Because of the high conservation of residues forming the small active site pocket among *T*. *cruzi* and human DHODHs, the design of a specific and potent inhibitor was difficult. Using the OF inducer approach, we obtained the OF structure of TcDHODH and showed that many residues surrounding the active site are specific for TcDHODH. By targeting the active site structure with OF, we show the first successful demonstration of the design of potent and selective inhibitors of a family 1A DHODH. The OF inducer approach will contribute to the development of new drugs against Chagas disease, and represents a powerful new tool for drug design. This approach has potential to encourage drug design against diseases where the molecular targets are such difficult proteins possessing small AS volume.

## Supporting Information

S1 Fig Molecular surfaces representation of the TcDHODH sites. (A) Without the effect of an open-form inducer (chain A of PDB ID 2E6F), (B) in the open form due to **1** interaction (chain A of PDB ID 3W1Q) and **c** chain B of PDB ID 3W1Q showing higher enlargement of active site due to additional effect of crystal packing. Protein backbone is represented as gray (chain A) and dark gray (chain B) ribbons with the residues L128-D142 (active loop) as red ribbon. Sites regions were identified using CASTp Server[56] and Figures produced with UCSF Chimera[57].
(TIF)

S2 Fig Surface representation of TcDHODH- 1 complex structure (3W1Q) showing the tunnel leading to the active site. The surrounding amino acid residues which become exposed by the active site loop movement are represented in stick. The movement of the active site loop make exposed hydrophobic region (formed by G70, L71, L101 and P131) and hydrogen bonding sites such as R50, N53, S68, S195, N198 and K214. Color codes are the same as Fig 2C except for the active site loop where carbon atoms were colored in orange. Electron density map, contoured at 1 σ, from Comp **1** is shown.
(TIF)

S3 Fig Predicted versus co-crystallized binding modes. Predicted and co-crystallized binding modes of **2** (A), **3** (B), **4** (C) and **5** (D) are shown in left and right panels, respectively. Hydrophobic interactions of 5-substituents with P131 (**2**–**5**) or G70 (**4**) are shown as dashed lines labeled by their distance (Å). Residues P131 and G70, **2**–**5** and FMN are represented by stick. Atoms of nitrogen and oxygen are colored in blue and red, respectively. Carbons from predicted and co-crystallized structures are shown in gray and salmon, respectively. Electron density map from **2**–**5** co-crystallized with TcDHODH (right panels) are shown as blue mesh and contoured at 1 σ level.
(TIF)

S4 Fig **Binding mode of 6 (A), 7 (B) and 8 (C) into chains A (left) and B (right).** Key residues within 4 Å distance to 5-subtituent groups, **6**, **7**, **8** and FMN are shown as stick. Dashed lines represent the hydrophobic or hydrogen bond interactions between 5-substitutent and protein residues with distances (Å) shown as number next to the respective interactions. Atoms of carbon, nitrogen and oxygen are colored in salmon, blue, and red, respectively. Electron density map of Comp **6**–**8** are shown as blue mesh and contoured at 1σ level.
(TIF)

S5 Fig Correlation between predicted docking score versus experimental IC_50_s for TcDHODH of 5-substituted orotate derivatives synthesized in this study. **1**, **5** and **9** were removed for better visualization of graph since their position overlaps with others.
(TIF)

S6 Fig Size exclusion chromatography of TcDHODH. The size of TcDHODH was estimated using TSK G3000SW (Tosoh) gel filtration column (7.5 × 600 mm). Purified TcDHODH was mixed with 50 ml Gel Filtration Standard (Bio-Rad) to a final concentration of 0.5 mg/ml and injected to the column. The analysis was performed at room temperature at flow rate of 1 ml/min in 100 mM sodium phosphate buffer pH 7.5, 150 mM NaCl and 0.25 mM sodium orotate. The molecular weight of TcDHODH was calculated based on the retention time of size markers that are: A-Void peak; B-Thyroglobulin (670 kDa); C-γ-globulin (158 kDa); D-Ovalbumin (44 kDa); E-Myoglobin (17 kDa) and F-Vitamin B12 (1.35 kDa). The black arrow indicates the peak corresponding to TcDHODH (37 kDa, as calculated from the standards).
(TIF)

S7 Fig DHODH activity staining of TcDHODH and HsDHODH. Left, blue native PAGE of TcDHODH followed by DHODH activity staining. 1250 to 20 ng of pure TcDHODH was applied onto 4–16% Bis-Tris gel (Invitrogen) and run at 150 V constant by light blue cathode buffer method (0.002% of G-250 blue dye) according to manufacturer’s instruction. Right, high resolution clear native electrophoresis of HsDHODH followed by DHODH activity staining. 1250 to 50 ng of purified HsDHODH was loaded onto 4–16% Bis-Tris gel (Invitrogen) and run at 150 V constant according to manufacturer instruction. The DHODH activity of TcDHODH and HsDHODH was stained as described in Material and Methods.
(TIF)

S1 Table Data collection and refinement statistics TcDHODH in complex with 5-substituted orotate derivatives. Values in parentheses are for the highest resolution shell. *, PF: Photon factory and SP8: SPring-8.
(DOCX)

S2 Table Data collection and refinement statistics of T. cruzi and human DHODHs in complex with compound 14. Values in parentheses are for the highest resolution shell. *, PF: Photon factory.
(DOCX)

S3 Table Dynamic light scattering analysis of rTcDHODH. ^a^, estimated molecular weight. For detailed Method, see reference [15] from main text.
(DOCX)
