# Supplementary figures and images for: The Open Form Inducer Approach for Structure-Based Drug Design
Source: PLoS One. 2016 Nov 28;11(11):e0167078. doi: 10.1371/journal.pone.0167078 (PMC5125662; doi:10.1371/journal.pone.0167078)

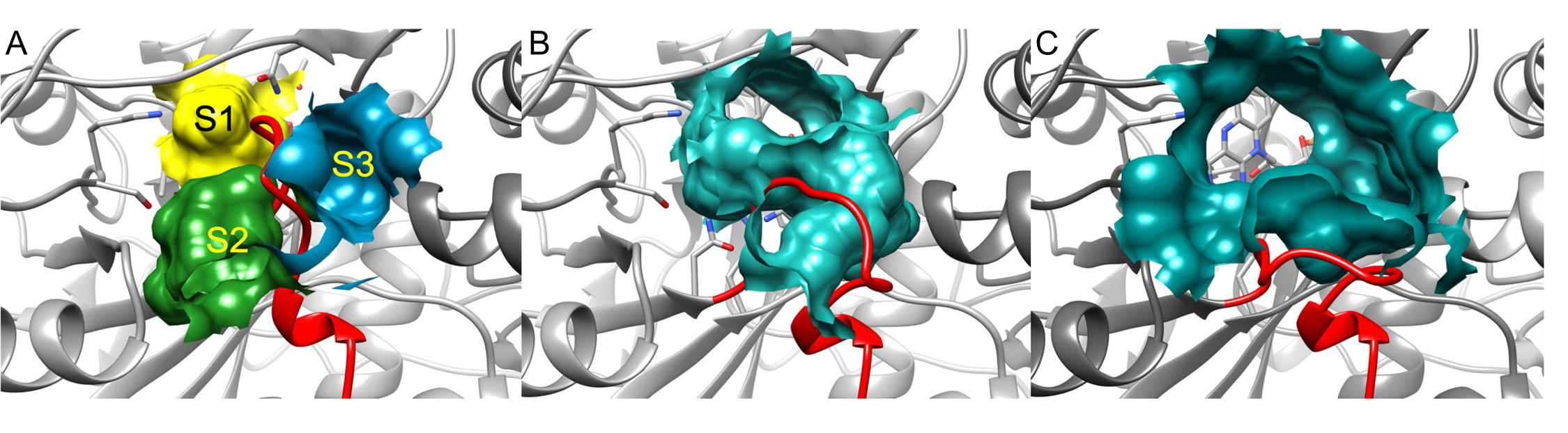

Supplement: S1 Fig — (A) Without the effect of an open-form inducer (chain A of PDB ID 2E6F), (B) in the open form due to 1 interaction (chain A of PDB ID 3W1Q) and c chain B of PDB ID 3W1Q showing higher enlargement of active site due to additional effect of crystal packing. Protein backbone is represented as gray (chain A) and dark gray (chain B) ribbons with the residues L128-D142 (active loop) as red ribbon. Sites regions were identified using CASTp Server[56] and Figures produced with UCSF Chimera[57]. (TIF) [file pone.0167078.s001.tif]

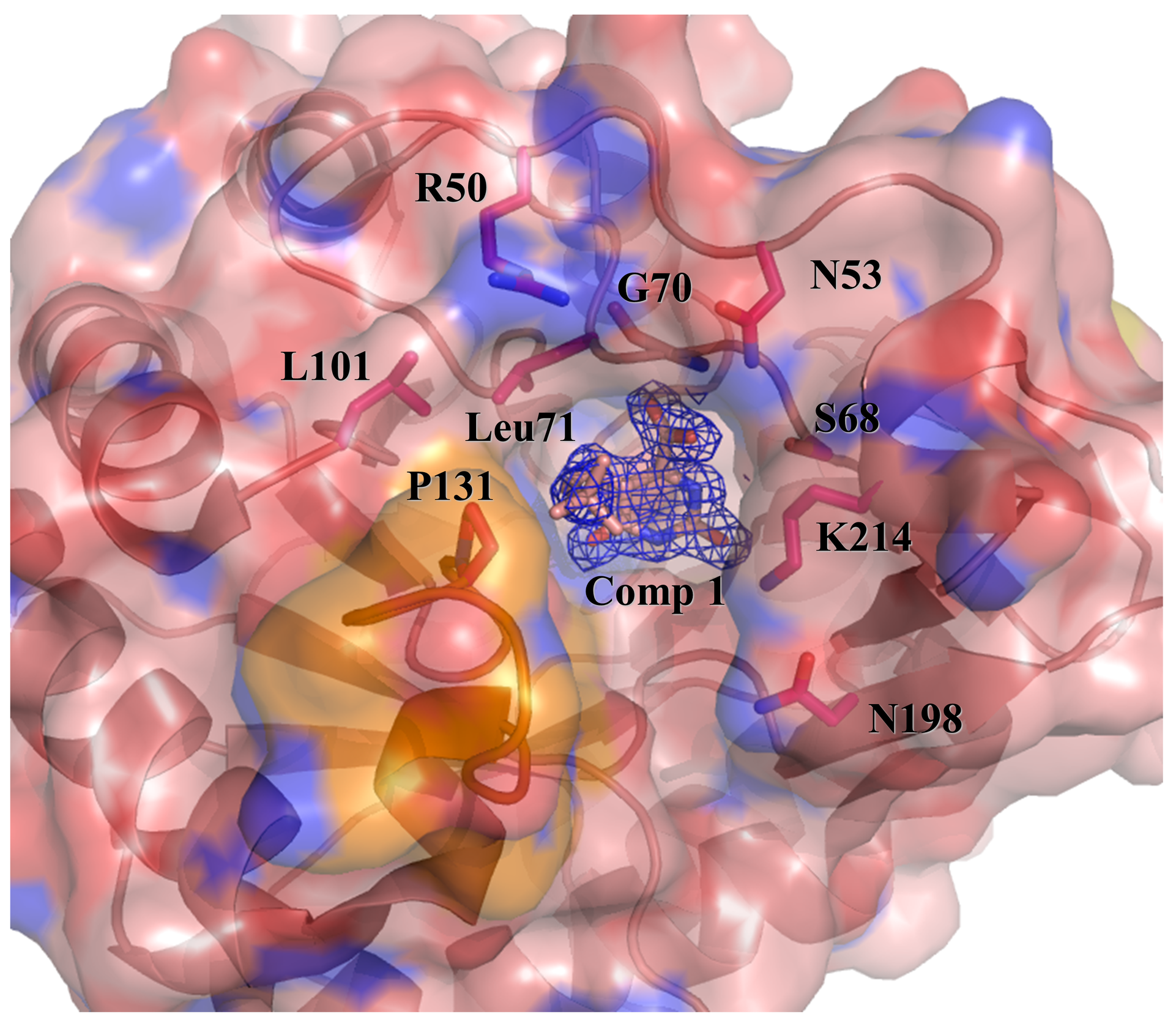

Supplement: S2 Fig — The surrounding amino acid residues which become exposed by the active site loop movement are represented in stick. The movement of the active site loop make exposed hydrophobic region (formed by G70, L71, L101 and P131) and hydrogen bonding sites such as R50, N53, S68, S195, N198 and K214. Color codes are the same as Fig 2C except for the active site loop where carbon atoms were colored in orange. Electron density map, contoured at 1 σ, from Comp 1 is shown. (TIF) [file pone.0167078.s002.TIF]

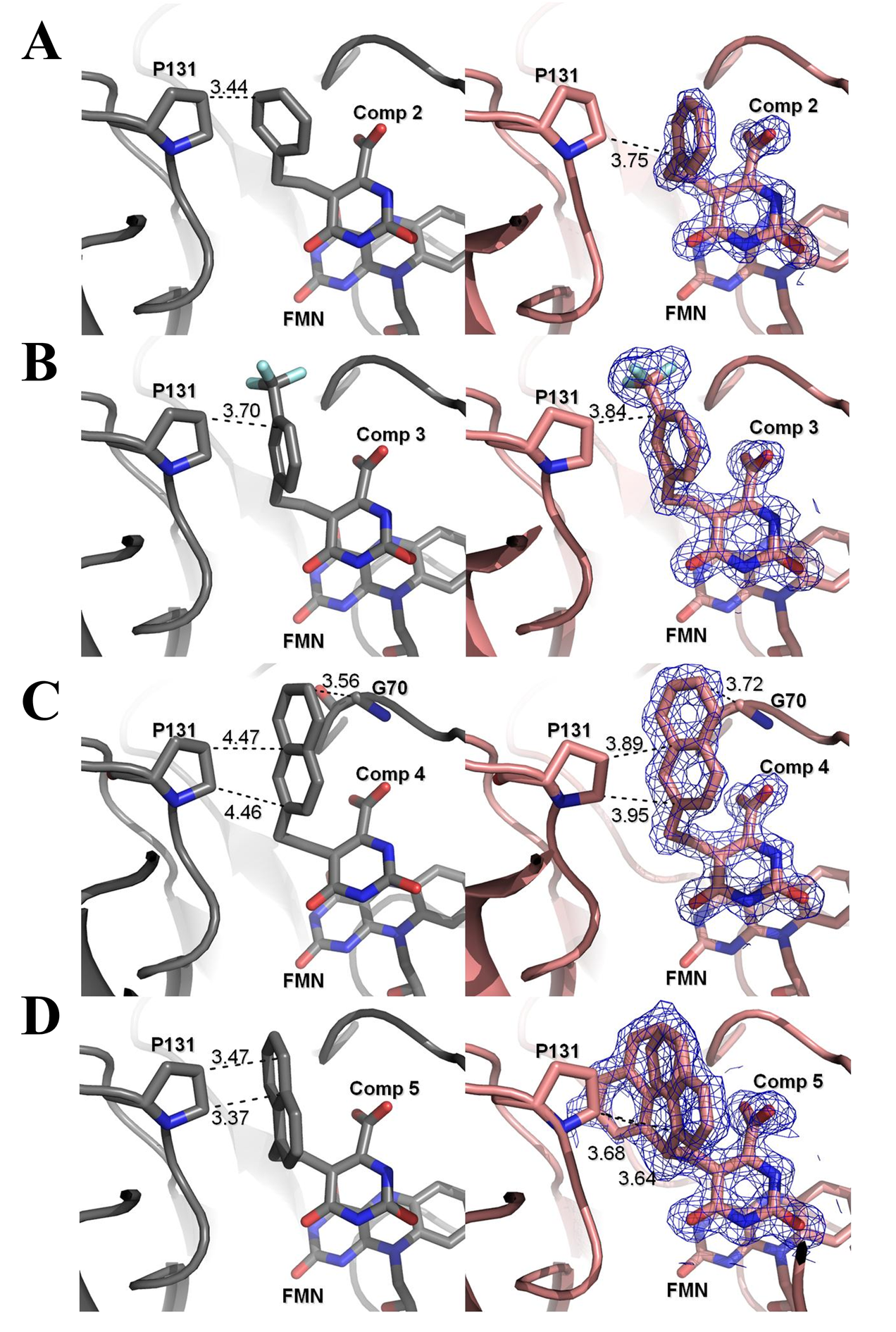

Supplement: S3 Fig — Predicted and co-crystallized binding modes of 2 (A), 3 (B), 4 (C) and 5 (D) are shown in left and right panels, respectively. Hydrophobic interactions of 5-substituents with P131 (2–5) or G70 (4) are shown as dashed lines labeled by their distance (Å). Residues P131 and G70, 2–5 and FMN are represented by stick. Atoms of nitrogen and oxygen are colored in blue and red, respectively. Carbons from predicted and co-crystallized structures are shown in gray and salmon, respectively. Electron density map from 2–5 co-crystallized with TcDHODH (right panels) are shown as blue mesh and contoured at 1 σ level. (TIF) [file pone.0167078.s003.TIF]

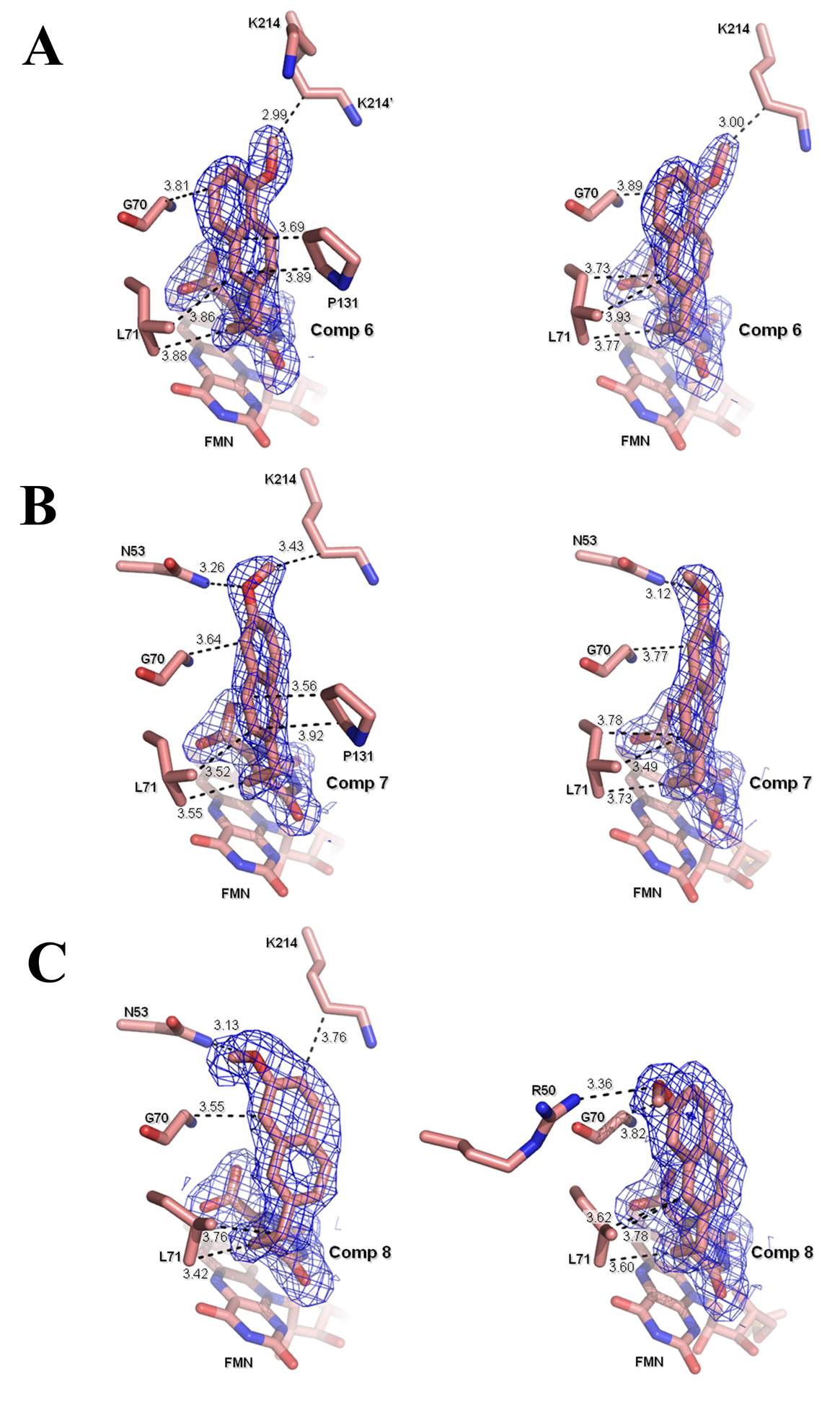

Supplement: S4 Fig — Binding mode of 6 (A), 7 (B) and 8 (C) into chains A (left) and B (right). Key residues within 4 Å distance to 5-subtituent groups, 6, 7, 8 and FMN are shown as stick. Dashed lines represent the hydrophobic or hydrogen bond interactions between 5-substitutent and protein residues with distances (Å) shown as number next to the respective interactions. Atoms of carbon, nitrogen and oxygen are colored in salmon, blue, and red, respectively. Electron density map of Comp 6–8 are shown as blue mesh and contoured at 1σ level. (TIF) [file pone.0167078.s004.TIF]

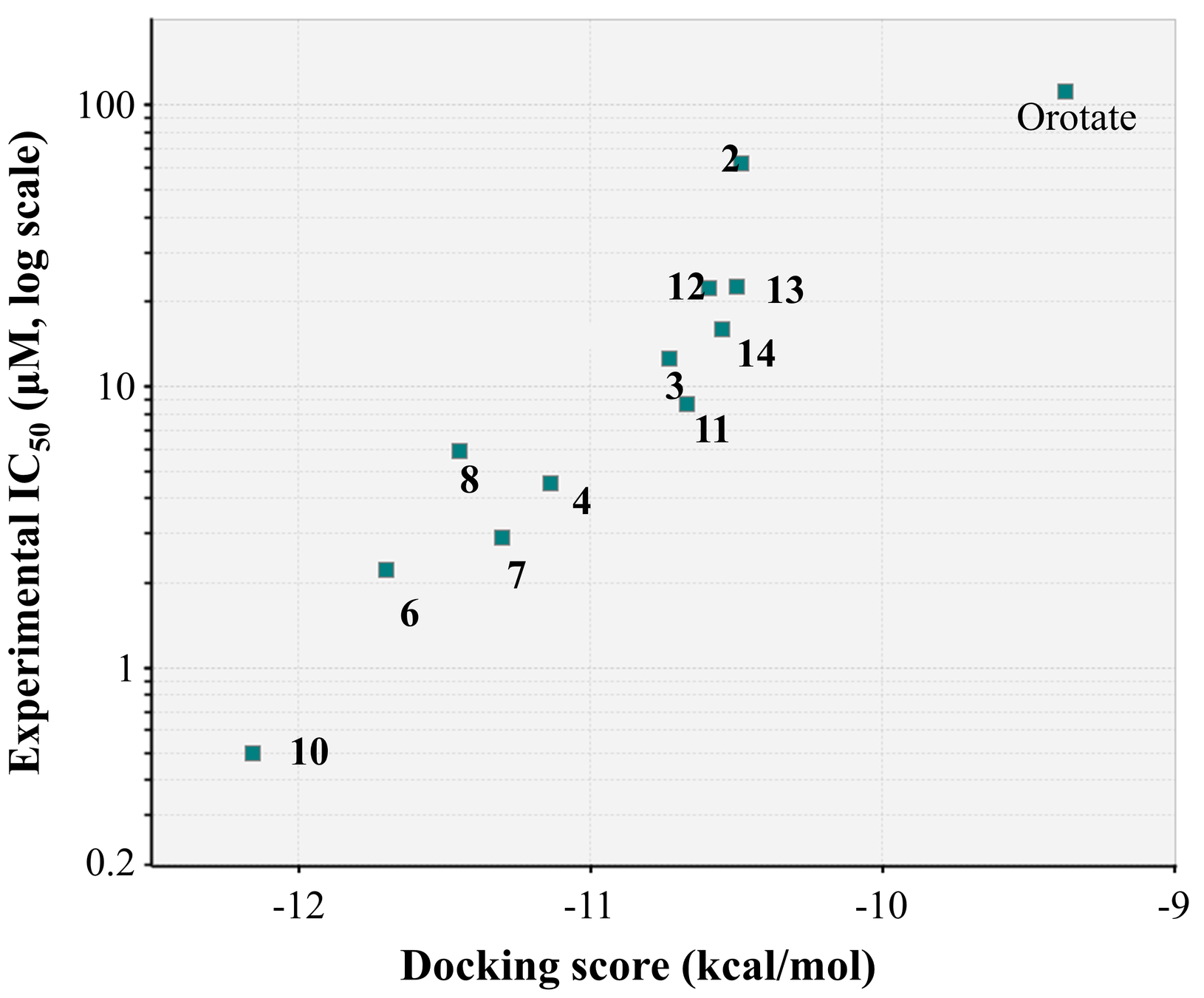

Supplement: S5 Fig — 1, 5 and 9 were removed for better visualization of graph since their position overlaps with others. (TIF) [file pone.0167078.s005.tif]

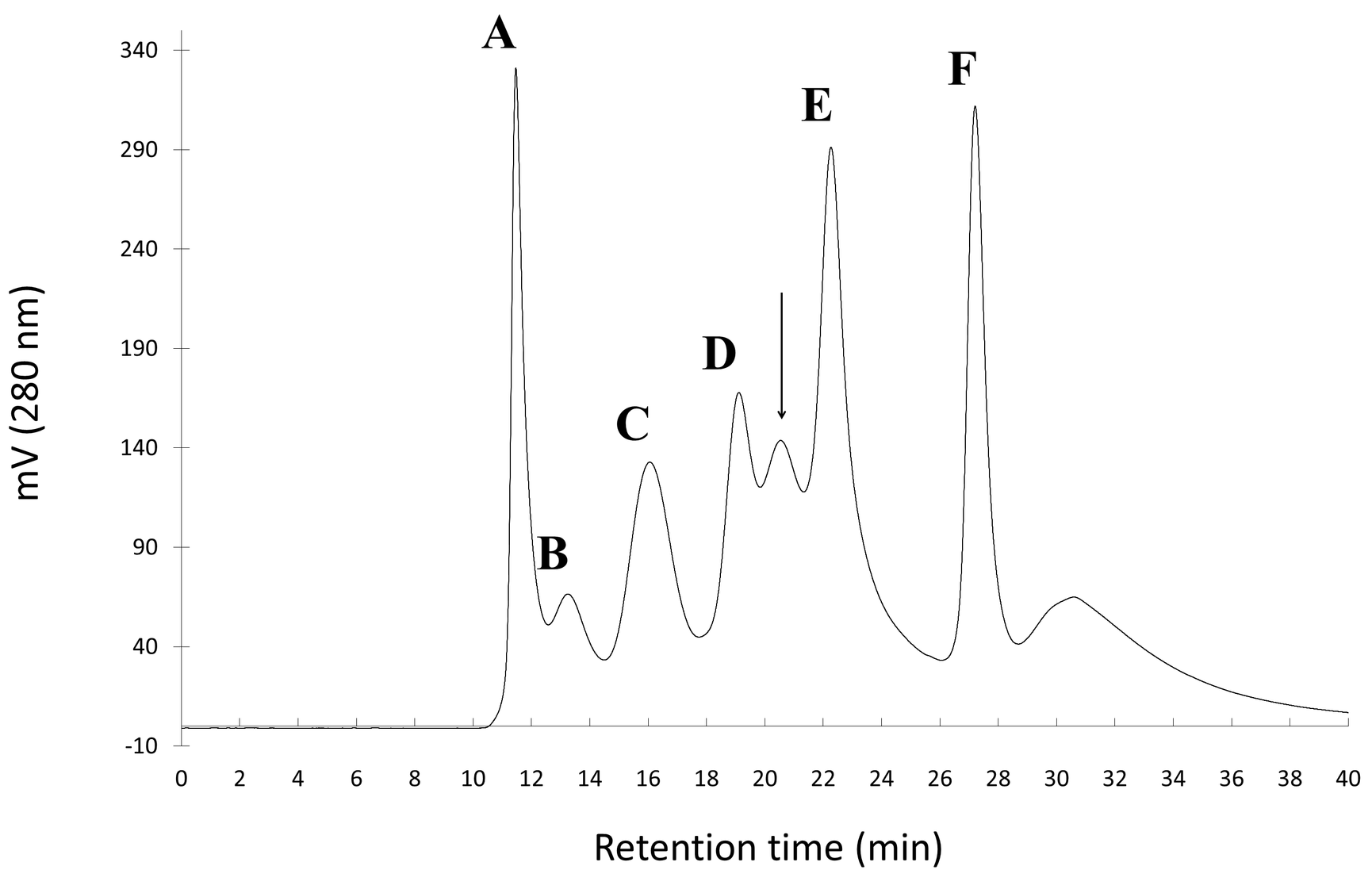

Supplement: S6 Fig — The size of TcDHODH was estimated using TSK G3000SW (Tosoh) gel filtration column (7.5 × 600 mm). Purified TcDHODH was mixed with 50 ml Gel Filtration Standard (Bio-Rad) to a final concentration of 0.5 mg/ml and injected to the column. The analysis was performed at room temperature at flow rate of 1 ml/min in 100 mM sodium phosphate buffer pH 7.5, 150 mM NaCl and 0.25 mM sodium orotate. The molecular weight of TcDHODH was calculated based on the retention time of size markers that are: A-Void peak; B-Thyroglobulin (670 kDa); C-γ-globulin (158 kDa); D-Ovalbumin (44 kDa); E-Myoglobin (17 kDa) and F-Vitamin B12 (1.35 kDa). The black arrow indicates the peak corresponding to TcDHODH (37 kDa, as calculated from the standards). (TIF) [file pone.0167078.s006.TIF]

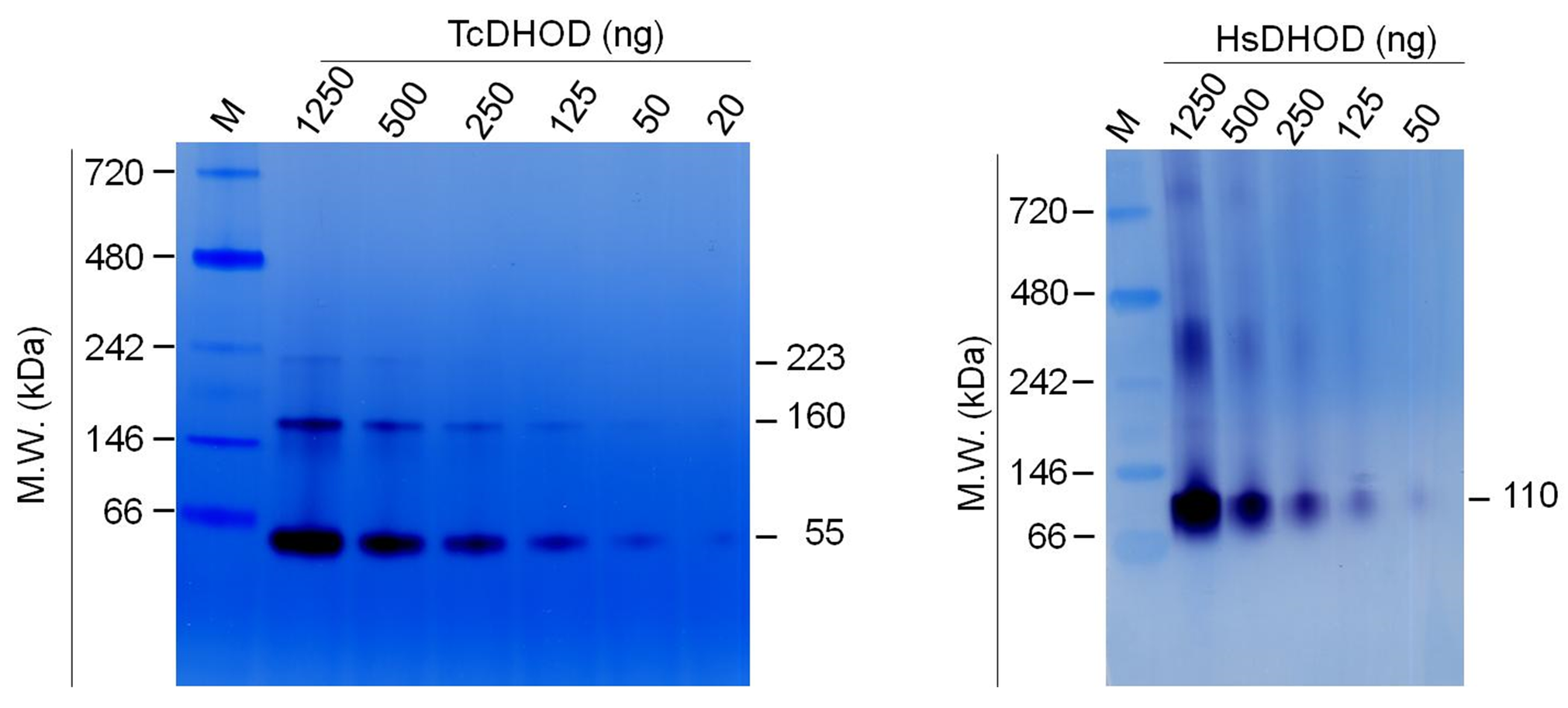

Supplement: S7 Fig — Left, blue native PAGE of TcDHODH followed by DHODH activity staining. 1250 to 20 ng of pure TcDHODH was applied onto 4–16% Bis-Tris gel (Invitrogen) and run at 150 V constant by light blue cathode buffer method (0.002% of G-250 blue dye) according to manufacturer’s instruction. Right, high resolution clear native electrophoresis of HsDHODH followed by DHODH activity staining. 1250 to 50 ng of purified HsDHODH was loaded onto 4–16% Bis-Tris gel (Invitrogen) and run at 150 V constant according to manufacturer instruction. The DHODH activity of TcDHODH and HsDHODH was stained as described in Material and Methods. (TIF) [file pone.0167078.s007.TIF]
