# Supplementary material for: The Open Form Inducer Approach for Structure-Based Drug Design
Source: PLoS One. 2016 Nov 28;11(11):e0167078. doi: 10.1371/journal.pone.0167078 (PMC5125662; doi:10.1371/journal.pone.0167078)
Supplement: S1 Table — Values in parentheses are for the highest resolution shell. *, PF: Photon factory and SP8: SPring-8. (DOCX) [file pone.0167078.s008.docx]

**S1 Table: Data collection and refinement statistics TcDHODH in complex with 5-substituted orotate derivatives.**

| Compound | **1** | **2** | **3** | **4** | **5** | **6** |
| --- | --- | --- | --- | --- | --- | --- |
| **Data collection** |  |  |  |  |  |  |
| Beam line* | PF, NW12A | PF, BL17A | PF, BL17A | PF, BL5A | PF, BL17A | SP8, BL44XU |
| Space group | *P*2_1_2_1_2_1_ | *P*2_1_2_1_2_1_ | *P*2_1_2_1_2_1_ | *P*2_1_2_1_2_1_ | *P*2_1_2_1_2_1_ | *P*2_1_2_1_2_1_ |
| Cell dimensions |  |  |  |  |  |  |
| *a*, *b*, *c* (Å) | 68.11, 71.89, 129.55 | 67.95, 71.81, 129.12 | 68.14, 71.91, 128.84 | 67.89, 71.71, 129.11 | 67.82, 71.88, 128.28 | 67.69, 71.45, 129.14 |
| Wavelength (Å) | 0.92 | 1.00 | 1.00 | 1.00 | 1.05 | 0.90 |
| Resolution (Å) | 50.0 - 1.85 | 50.0 - 1.58 | 50.0 - 1.68 | 50.0 - 1.67 | 50.0 - 1.55 | 50.0 - 1.75 |
|  | (1.92 - 1.85) | (1.64 - 1.58) | (1.74 - 1.68) | (1.70 - 1.67) | (1.58 - 1.55) | (1.78 - 1.75) |
| *R*_merge_ (%) | 6.7 (25.1) | 8.7 (42.2) | 7.2 (41.1) | 6.8 (43.8) | 6.6 (40.9) | 7.2 (40.8) |
| *I* / σ(*I*) | 15.0 (7.66) | 9.50 (3.39) | 9.9 (2.95) | 13.1 (5.57) | 13.6 (3.36) | 12.5 (4.23) |
| Completeness (%) | 96.7 (92.9) | 98.8 (97.5) | 88.0 (87.2) | 99.9 (100) | 98.9 (99.5) | 76.6 (77.2) |
| Redundancy | 6.0 | 6.1 | 5.7 | 6.7 | 4.3 | 4.4 |
|  |  |  |  |  |  |  |
| **Refinement** |  |  |  |  |  |  |
| Resolution (Å) | 34.6 - 1.85 | 46.1 - 1.58 | 34.1 - 1.68 | 29.9 - 1.67 | 39.1 - 1.55 | 34.0 - 1.75 |
| No. reflections | 50236 | 81405 | 60663 | 70491 | 86300 | 47115 |
| *R*_work_ / *R*_free_ | 0.15 / 0.19 | 0.16 / 0.18 | 0.14 / 0.18 | 0.14/ 0.18 | 0.14/ 0.17 | 0.14 / 0.19 |
| No. atoms |  |  |  |  |  |  |
| Protein  FMN | 4776  31 | 4776  31 | 4776  62 | 4830  62 | 4776  62 | 4796  62 |
| Ligand | 34 | 38 | 46 | 69 | 68 | 50 |
| Water | 559 | 675 | 632 | 631 | 700 | 594 |
| *B*-factors |  |  |  |  |  |  |
| Protein |  |  |  |  |  |  |
| A chain | 14.2 | 11.0 | 12.2 | 14.4 | 14.9 | 12.9 |
| B chain | 13.2 | 11.9 | 13.1 | 15.9 | 14.1 | 15.1 |
| FMN | 6.9 | 6.3 | 7.5 | 9.3 | 9.6 | 7.6 |
| Ligand | 16.0 | 12.9 | 15.0 | 18.9 | 11.5 | 14.7 |
| Water | 23.3 | 24.3 | 25.0 | 28.3 | 29.9 | 26.2 |
| R.m.s. deviations |  |  |  |  |  |  |
| Bond Lengths (Å) | 0.02 | 0.03 | 0.02 | 0.02 | 0.03 | 0.02 |
| Bond angles (º) | 2.01 | 2.46 | 2.09 | 2.26 | 2.41 | 1.97 |
|  |  |  |  |  |  |  |
| **PDB ID** | 3W1Q | 3W1R | 3W1T | 3W7H | 3W72 | 3W7C |

| Compound | **7** | **8** | **9** | **10** | **11** | **12** |
| --- | --- | --- | --- | --- | --- | --- |
| **Data collection** |  |  |  |  |  |  |
| Beam line* | SP8, BL44XU | PF, BL5A | PF, NE3A | SP8, BL44XU | PF, BL5A | PF, BL17A |
| Space group | *P*2_1_2_1_2_1_ | *P*2_1_2_1_2_1_ | *P*2_1_2_1_2_1_ | *P*2_1_2_1_2_1_ | *P*2_1_2_1_2_1_ | *P*2_1_2_1_2_1_ |
| Cell dimensions |  |  |  |  |  |  |
| *a*, *b*, *c* (Å) | 67.99, 71.53, 129.37 | 67.93, 71.64, 129.18 | 67.88, 71.57, 129.24 | 68.00, 71.69, 129.42 | 67.92, 71.74, 129.02 | 68.06, 71.64, 129.29 |
| Wavelength (Å) | 0.90 | 1.00 | 1.00 | 0.90 | 1.00 | 1.00 |
| Resolution (Å) | 50.0 - 1.97 | 50.0 - 1.82 | 50.0 - 2.63 | 50.0 - 1.58 | 50.0 - 1.85 | 50.0 - 1.98 |
|  | (2.00 - 1.97) | (1.85 - 1.82) | (2.68 - 2.63) | (1.61 - 1.58) | (1.92 - 1.85) | (2.05 - 1.98) |
| *R*_merge_ (%) | 8.4 (39.8) | 8.5 (43.6) | 14.0 (42.5) | 9.3 (41.8) | 8.5 (43.2) | 8.6 (40.7) |
| *I* / σ(*I*) | 9.7 (5.04) | 10.4 (5.20) | 5.5 (2.83) | 10.3 (5.00) | 8.9 (4.75) | 6.3 (3.41) |
| Completeness (%) | 99.2 (99.4) | 99.5 (100) | 93.9 (95.8) | 91.2 (89.8) | 100 (100) | 95.6 (94.2) |
| Redundancy | 5.6 | 7.0 | 4.5 | 5.5. | 7.1 | 5.6 |
|  |  |  |  |  |  |  |
| **Refinement** |  |  |  |  |  |  |
| Resolution (Å) | 27.5 - 1.97 | 32.8 - 1.82 | 39.3 - 2.68 | 39.3 - 1.58 | 26.6 - 1.85 | 47.99 - 1.98 |
| No. reflections | 43256 | 54089 | 17040 | 76157 | 51868 | 40834 |
| *R*_work_ / *R*_free_ | 0.15 / 0.19 | 0.17 / 0.21 | 0.19 / 0.28 | 0.15 / 0.18 | 0.15 / 0.18 | 0.16 / 0.21 |
| No. atoms |  |  |  |  |  |  |
| Protein  FMN | 4776  62 | 4776  62 | 4776  62 | 4782  62 | 4776  62 | 4776  62 |
| Ligand | 50 | 50 | 52 | 78 | 40 | 46 |
| Water | 417 | 532 | 257 | 608 | 596 | 412 |
| *B*-factors |  |  |  |  |  |  |
| Protein |  |  |  |  |  |  |
| A chain | 19.0 | 19.8 | 22.3 | 9.8 | 15.2 | 22.9 |
| B chain | 21.1 | 21.8 | 23.6 | 11.8 | 16.5 | 24.5 |
| FMN | 12.7 | 14.6 | 16.4 | 5.2 | 10.0 | 15.1 |
| Ligand | 19.8 | 26.2 | 40.6 | 10.3 | 18.8 | 26.8 |
| Water | 28.4 | 31.6 | 18.0 | 23.6 | 26.2 | 30.4 |
| R.m.s. deviations |  |  |  |  |  |  |
| Bond Lengths (Å) | 0.02 | 0.02 | 0.01 | 0.02 | 0.02 | 0.02 |
| Bond angles (º) | 2.01 | 2.07 | 1.54 | 2.32 | 2.09 | 1.95 |
|  |  |  |  |  |  |  |
| **PDB ID** | 3W3O | 4JDB | 3W6Y | 3W7J | 3W1U | 3W22 |

| Compound | **13** |
| --- | --- |
| **Data collection** |  |
| Beam line* | PF, BL17A |
| Space group | *P*2_1_2_1_2_1_ |
| Cell dimensions |  |
| *a*, *b*, *c* (Å) | 67.99, 71.85, 128.64 |
| Wavelength (Å) | 1.00 |
| Resolution (Å) | 50.0 - 2.60 |
|  | (2.64 - 2.60) |
| *R*_merge_ (%) | 12.2 (37.9) |
| *I* / σ(*I*) | 9.7 (7.23) |
| Completeness (%) | 87.5 (93.3) |
| Redundancy | 5.0 |
|  |  |
| **Refinement** |  |
| Resolution (Å) | 46.8 - 2.60 |
| No. reflections | 16117 |
| *R*_work_ / *R*_free_ | 0.20/ 0.27 |
| No. atoms |  |
| Protein  FMN | 4776  62 |
| Ligand | 52 |
| Water | 282 |
| *B*-factors |  |
| Protein |  |
| A chain | 24.6 |
| B chain | 25.7 |
| FMN | 21.4 |
| Ligand | 42.5 |
| Water | 24.0 |
| R.m.s. deviations |  |
| Bond Lengths (Å) | 0.01 |
| Bond angles (º) | 1.55 |
|  |  |
| **PDB ID** | 3W70 |

Values in parentheses are for the highest resolution shell. *, PF: Photon factory and SP8: SPring-8
