# Supplementary material for: The Open Form Inducer Approach for Structure-Based Drug Design
Source: PLoS One. 2016 Nov 28;11(11):e0167078. doi: 10.1371/journal.pone.0167078 (PMC5125662; doi:10.1371/journal.pone.0167078)
Supplement: S2 Table — Values in parentheses are for the highest resolution shell. *, PF: Photon factory. (DOCX) [file pone.0167078.s009.docx]

**S2 Table: Data collection and refinement statistics of *T. cruzi* and human DHODHs in complex with compound 14.**

| Compound | **14** | |
| --- | --- | --- |
| DHODH | *T. cruzi* | Human |
| **Data collection** |  |  |
| Beam line* | PF, BL17A | PF, BL17A |
| Space group | *P*2_1_2_1_2_1_ | *P*3_2_21 |
| Cell dimensions |  |  |
| *a*, *b*, *c* (Å) | 68.11, 71.81, 129.18 | 90.45, 90.45, 123.06 |
| Wavelength (Å) | 1.00 | 0.98 |
| Resolution (Å) | 50.0 -1.68 | 50.0 - 1.68 |
|  | (1.71 - 1.68) | (1.71 - 1.68) |
| *R*_merge_ (%) | 7.1 (42.4) | 5.8 (41.0) |
| *I* / σ(*I*) | 16.2 (5.39) | 11.5 (6.31) |
| Completeness (%) | 99.4 (99.1) | 100 (100) |
| Redundancy | 5.8 | 11.0 |
| **Refinement** |  |  |
| Resolution (Å) | 39.2 - 1.68 | 36.3 - 1.68 |
| No. reflections | 68616 | 63486 |
| *R*_work_ / *R*_free_ | 0.14 / 0.17 | 0.14 / 0.17 |
| No. atoms |  |  |
| Protein  FMN | 4776  62 | 2944  31 |
| Ligand | 50 | 50 |
| Water | 624 | 319 |
| *B*-factors |  |  |
| Protein |  |  |
| A chain | 15.2 | 16.8 |
| B chain | 16.6 |  |
| FMN | 9.6 | 7.0 |
| Ligand | 21.3 | 16.1 |
| Water | 30.6 | 32.7 |
| R.m.s. deviations |  |  |
| Bond Lengths (Å) | 0.02 | 0.01 |
| Bond angles (º) | 2.22 | 1.71 |
|  |  |  |
| **PDB ID** | 3W71 | 3W7R |

Values in parentheses are for the highest resolution shell. *, PF: Photon factory.
