# Supplementary material for: The Open Form Inducer Approach for Structure-Based Drug Design
Source: PLoS One. 2016 Nov 28;11(11):e0167078. doi: 10.1371/journal.pone.0167078 (PMC5125662; doi:10.1371/journal.pone.0167078)
Supplement: S3 Table — a, estimated molecular weight. For detailed Method, see reference [15] from main text. (DOCX) [file pone.0167078.s010.docx]

**S3 Table: Dynamic light scattering analysis of rTcDHODH.** ^a^, estimated molecular weight. For detailed Method, see reference [15] from main text.

| TcDHODH (mg/ml) | 4.0 | 2.5 | 0.64 | 0.34 | 0.25 |
| --- | --- | --- | --- | --- | --- |
| Rh radius (nm) | 5.64 | 5.27 | 4.00 | 3.82 | 3.91 |
| M.W.^a^ (kDa) | 96.4 | 81.6 | 42.0 | 37.5 | 39.6 |
| Oligomerization | Dimer | Dimer | Monomer | Monomer | Monomer |
